# Automated pipette failure monitoring using image processing for point-of-care testing devices

**DOI:** 10.1186/s12938-018-0578-1

**Published:** 2018-11-06

**Authors:** Chan-Young Park, Jun Yeon, Hye-Jeong Song, Yu-Seop Kim, Ki-Bong Nahm, Jong-Dae Kim

**Affiliations:** 1 Department of Convergence Software, Hallym University, Chuncheon, South Korea; 2 Bio-IT Research Center, Hallym University, Chuncheon, South Korea; 3 Department of Electron Physics, Hallym University, Chuncheon, South Korea

**Keywords:** Automated liquid handler, Image processing, Pipetting failure, Projection

## Abstract

**Background:**

The accuracy and precision of liquid handling can be altered by several causes including wearing or failure of parts, and human error. The last cause is crucial since point-of-care testing (POCT) devices can be used by non-experienced users or patients themselves. Therefore it is important to improve the method of informing the users of POCT device malfunctions due to damage of parts or human error.

**Methods:**

In this paper, image-based failure monitoring of the automated pipetting was introduced for POCT devices. An inexpensive, high-performance camera for smartphones was employed in our previous work to resolve various malfunctions such as incorrect insertion of the tip, false positioning of the tip and pump, and improper operation of the pump. The image acquired from the camera was analyzed to detect the malfunctions. In this paper, the reagent volume in the tip was estimated from the image processing to verify the pump operation. First, the color component corresponding to the reagent intrinsic color was extracted to identify the reagent area in the tip before applying the binary image processing. The extracted reagent area was projected horizontally and the support length of the projection image was calculated. As the support length was related to the reagent volume, it was referred to the volume length. The relationship between the measured volume length and the previously measured solution mass was investigated. If we can predict the mass of the solution by the volume length, we will be able to detect the pump malfunction.

**Results:**

The cube of the volume length obtained by the proposed image processing method showed a very linear relationship with the reagent mass in the tip injected by the pumping operation (R^2^ = 0.996), indicating that the volume length could be utilized to estimate the reagent volume to monitor the accuracy and precision of the pumping operation.

**Conclusions:**

An inexpensive smartphone camera was enough to detect various malfunctions of a POCT device with pumping operation. The proposed image processing could monitor the level of inaccuracy of pumping volume in limited range. The simple image processing such as a fixed threshold and projections was employed for the cost optimization and system robustness. However it delivered the promising results because the imaging condition was highly controllable in the devices.

## Background

Liquid handling is an important part of many experiments in fields related to biology and chemistry, and is frequently used in genomic or proteomic research. In general, liquid handling used in such experiments requires accuracy and precision, but is also a very tedious task requiring a considerable amount of time if carried out manually [1–8]. Therefore, researches on automation of liquid handling have become paramount [1, 3, 5–7]. Most automated liquid handler (ALH) systems use a conveyor belt to optimize fast inspection of multiple samples at a large hospital. However, such systems require a complicated set of equipment for diagnosis and analysis, and hence can only be properly installed in large central laboratories that has access to various devices. Therefore it is difficult to implement the ALH system in developing countries where lack of central laboratories. Even in developed countries, small hospitals find it difficult to apply this system, resulting in manual clinical tests. As an alternative, small hospitals may send samples to a central laboratory equipped with the appropriate system, but this will result in patients feeling uncomfortable due to the slow turnaround time of the test results. To address these problems, a portable clinical test system using robotic automation was recently developed [4]. Despite the throughput might be smaller than that of a conventional system, it is relatively flexible, small, and inexpensive. Moreover, such a point-of-care test (POCT) device performs the task of a portable clinical test while being suitable to carry out the selective diagnosis work required at small hospitals [9–17]. Aided by the development of lab-on-a-chip (LOC) techniques, the development of POCT devices are also accelerating [10, 13–15, 17].

Volume verification using gravimetric, fluorescence, photometric and other methods is an important issue for accurate and precise liquid handling [18]. However, improvements in contactless dispensing technology using solenoid, piezoelectric, acoustic, and pneumatic systems provide high accuracy and precision [7]. Therefore, it is more important to monitor the aging or failure of equipment components from the point of view of human factor engineering rather than the problem of volume verification [19, 20]. Particularly, since POCT devices are used by inexperienced users or patients themselves, it is important to solve machine and human errors [9, 11]. As the development of POCT devices accelerates, non-professional users will increase. It is therefore important to improve the way users are informed of POCT device malfunctions due to component damage or human error [18, 19]. Multiple sensors should be used to provide users with informative functions for various malfunctions. However, as suggested in this study and our previous work, it is very useful to detect many malfunctions with a low cost camera.

In this paper, a new image-based monitoring method of the automated pipetting is introduced for POCT devices. Many devices require verification of device malfunction or user mistakes in addition to volume verification. In automated pipetting systems with a tip and a pump, various malfunctions must to be checked, such as incorrect insertion of the tip, false positioning of the tip and pump, and improper operation of the pump. A relatively simple and low-cost image processing system to conduct such validations can be implemented in ALH equipment by employing the recently developed inexpensive, high-performance cameras for smartphones. Most of the systems developed recently are equipped with graphical user interface which can provide users with information intuitively. Therefore taking benefit of the graphical user interface, the low-cost image processing system can achieve the superior usability, efficiency, and user satisfaction. Especially, users can check the operation status or identify what is malfunctioning while actually observing the internal situation of the ALH system [19].

Since the camera position is fixed and the lighting condition can be known in advance, a stable function can be implemented without any algorithmic error when an image is converted into a binary image with a fixed threshold value or even when a simple projection technique is used. In our previous work, the projection analysis of the captured image was successfully employed for robust identification of the tip and holder area, detecting absence and false positioning of the tip and the holder [21]. In the work, a simple fixed threshold and support analysis of the horizontal and vertical projection were enough to achieve zero failure rate. The tip position was detected with the support center of the horizontal projection, and the vertical position of the tip holder was identified by the upper limit of the support of the vertical projection.

In this study, we focus on the monitoring of the pumping operation. The accuracy and precision of pumping operation is first analyzed with a chemical balance. The volume lengths obtained from the image processing are compared with the pumping steps and they are proved to be utilized to detect the malfunction of the pumping operation. It is also given that the volume length can be utilized to estimate the reagent mass from the experimental results on the accuracy and precision of the pumping operation. Basically simple image processing techniques such as a fixed threshold and projections are preferred again as in our previous work for the cost optimization and system robustness [21].

The rest of this paper is organized as follows: We describe the materials and methods in “Methods” section, the results and the discussion are presented in “Results” and “Discussions” sections, respectively, and the conclusion is followed in “Conclusions” section.

## Methods

**Fig. 1:**
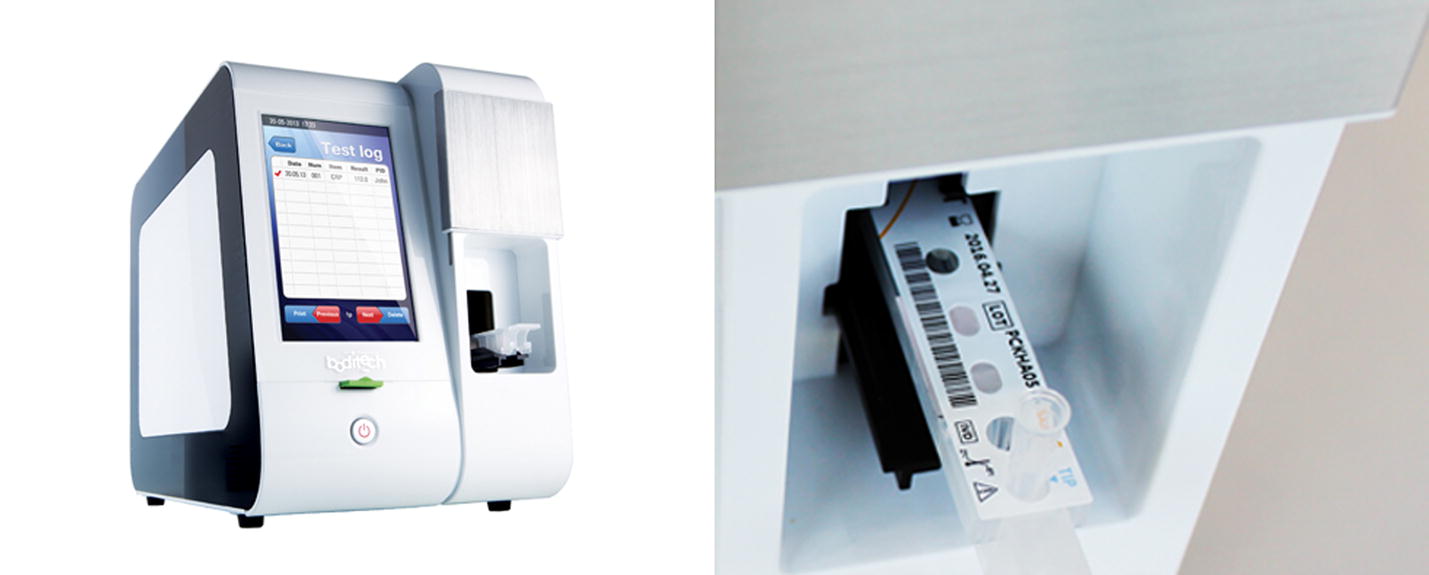
IChroma™ Smart Reader (left) and the cartridge loading example (right)

**Fig. 2:**
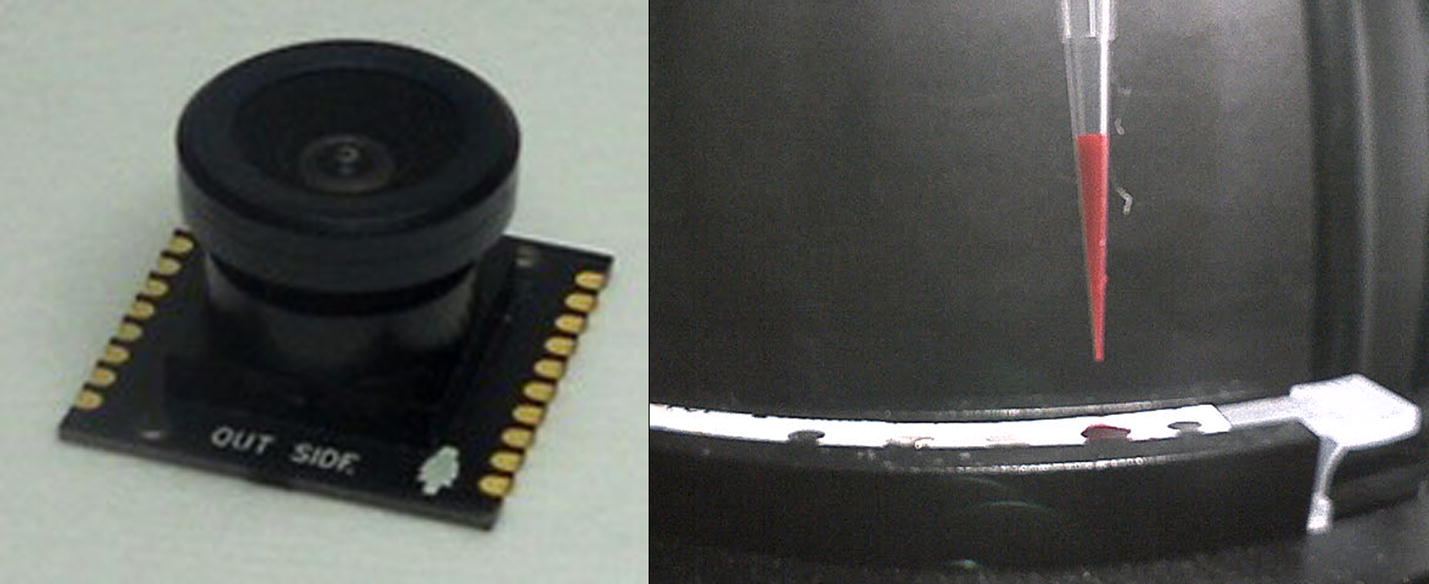
PO1150K Smart Camera module (left) and the image of the tube loaded with reagent (right)

A commercial automated system (IChroma™ Smart Reader, Boditech Med Inc.) shown in Fig. 1 was modified for the experiments in this paper. Before starting the analysis, the cartridge is inserted in the middle right part of the equipment shown in the image to the left of the figure. The cartridge contains the samples to be analyzed and the various reagents required. The left image of the figure is the enlarged view of the cartridge mounting part. As you can see in this figure, a pipette tip is put together with the cartridge. This device measures the concentration of the target substance in blood or blood serum of specimens including human, by using a specially designed cartridge. For the diagnosis, a pipette tip should be inserted manually in addition to the cartridge. The tip and the tip holder are the most important components in the device, and any malfunction of those components will lead to an inaccurate diagnosis result. After mounting the reagent and tip in the cartridge, the user inserts the cartridge in the device and presses the start button to initiate the diagnostic analysis. The tip is first mounted onto the tip holder of the pump installed within the device, and the reagents are pumped in/out to/from various chambers in the cartridge. For an accurate diagnosis, malfunctions such as the absence of the tip, misalignment of the tip and holder, and inaccurate amount of the reagent loaded into the tip must be identified and resolved during the diagnosis process. To perform these checks, the smartphone camera (PO1150K, Pixelplus, Co., Ltd.) was mounted in such a way that the tip holder of the pump would be positioned at the middle of the horizontal axis in the image. The employed camera shown in the left of Fig. 2 is an inexpensive module commonly used in smartphones. The right image of the figure shows the example captured using the camera.

The image processing functions are implemented in the host Android platform, where the LCD and touch panel are located in front of the device as shown in the left image of Fig. 1. Simple algorithms are preferred considering the computational power of the host. In our previous work, the binarization with a fixed threshold and the projections of the binary image were sufficient to successfully detect the absence of the tip or the holder. In this work, we focus on the monitoring of the pump operation.

First, the relation between the pump steps and the reagent mass loaded to the tip was analyzed in order to verify the accuracy and precision of the pump. The reagent mass loaded were measured every 40 steps from 0 to 640 steps with a chemical balance (ARG222, Ohaus Corp., USA). The loading and measurement for precision testing were repeated 5 times for each step. The mass of the tip and tip cover were measured before loading and subtracted from the total mass of the loaded tip and tip cover. The tip and the cover were changed for every measurements because it is difficult to remove the reagent totally from the tip. The loaded tip was imaged before being covered and unmounted from the holder to measure the mass.

**Fig. 3:**
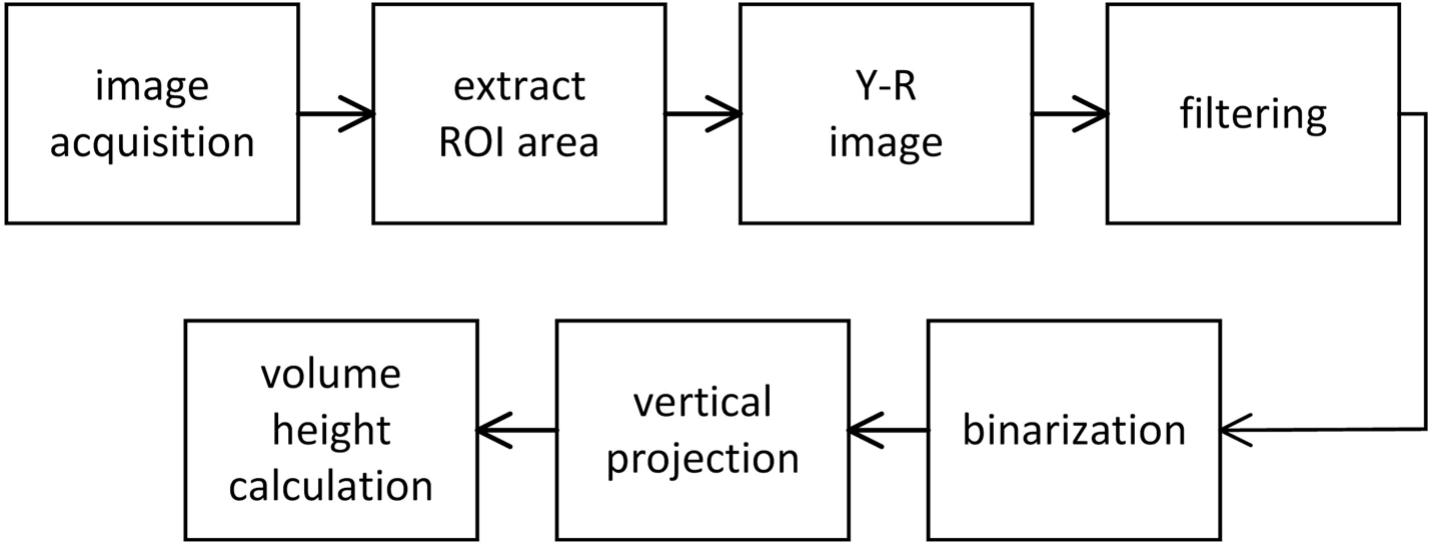
Schematic of flow algorithm for image processing

**Fig. 4:**
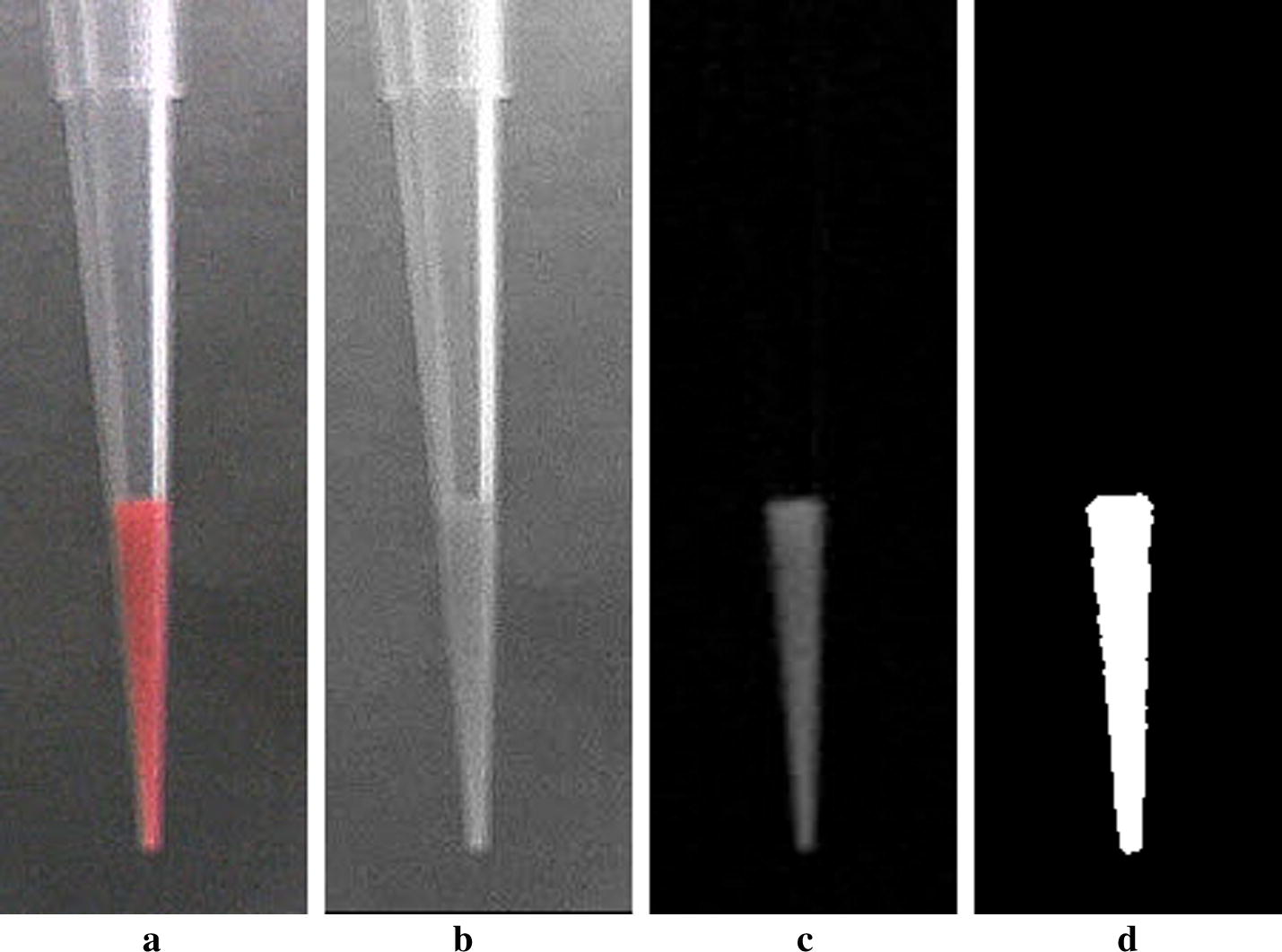
**a** ROI of entire tip image; **b** the luminance image; **c** the pure red image; **d** the extracted reagent area

Figure 3 shows the whole flow of the image processing for pipette monitoring. Given that the horizontal axis position of the tip holder is constant, the region of interest (ROI) was set centered to that position for the image acquired. Figure 4a shows an example of ROI images. Since red-colored water was used to emulate the blood samples in this study, the reagent area was extracted from the pure red image (Y-R image block in Fig. 3). The pure red image was obtained by subtracting the red channel to the luminance image (Fig. 4b, c). It was binarized with a fixed threshold after 3 × 3 median filtering. Binarization with a fixed threshold was sufficient as in our previous work, because the imaging condition such as illumination and background matte finish could be sufficiently controlled over production.

**Fig. 5:**
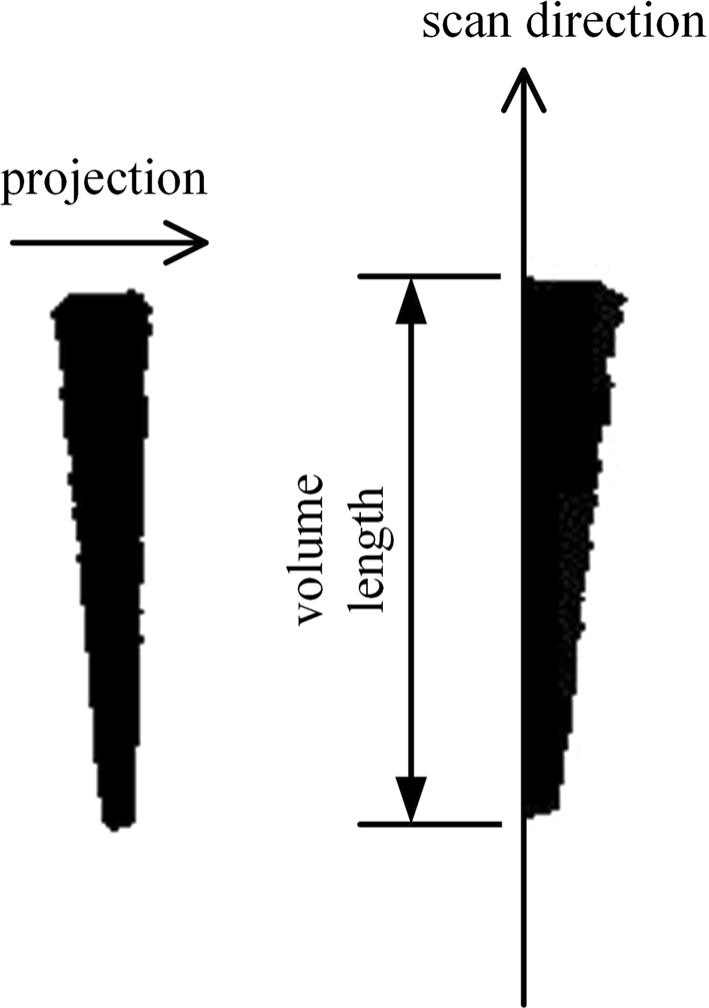
The horizontal projection of the reagent area (left) and the volume length defined on the projection image (right)

The extracted reagent area shown in the left of Fig. 5 was projected horizontally and the resultant projection image in the right of the figure was analysed. The support length of the projection image was calculated by scanning the projection vertically and counting the non-zero valued vertical positions. As the support length was related to the reagent volume, the support length was referred to the volume length as shown in the right image of Fig. 5. Note that the actual reagent volume was proportional to the cube of the volume length because of the conical shape of the tip. Various sophisticated methods for binary image filtering and calculation of the projection support can be applicable if the above simple procedure cannot achieve correct operation. However, the proposed simple procedure alone was sufficient for our experiments.

## Results

**Fig. 6:**
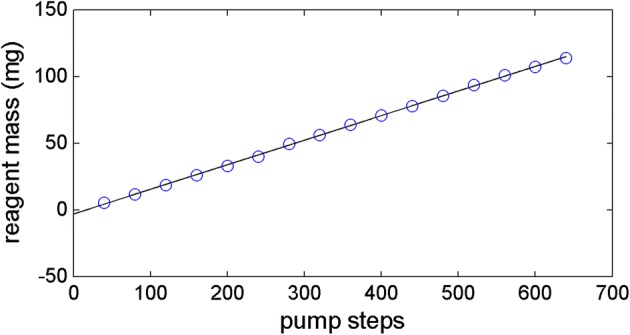
The linearity between the number of pump steps and average reagent mass (fitted linear equation: *y* = 18.4*x* + 0.001, *R*^2^ = 1.000)

The average and standard deviation of the reagent mass for each step is shown in Table 1, where the coefficients of variation (CV) values are shown in the last row. The CV values were less than 5.7 ppm, indicating that the pump loaded the precise amount of reagents. By regression analysis, the CV values were not related to the steps showing that the precision was independent of the pump steps. The coefficient of determinant (R^2^) which represents the linearity between the average masses and pump steps was ‘1’ as shown in Fig. 6, verifying the accuracy of the pump.

**Table 1:** The linearity between the number of motor steps and reagent mass (five experiments per each number of steps)

Steps	40	80	120	160	200	240	280	320
Mean	5.28	11.46	18.66	25.80	32.80	39.94	49.54	56.08
STD	0.26	0.34	0.94	0.82	1.04	2.28	1.50	2.04
CV (ppm)	4.9	2.9	5.0	3.2	3.2	5.7	3.0	3.6

Steps	360	400	440	480	520	560	600	640
Mean	63.68	70.70	77.64	85.52	93.48	100.98	107.14	113.72
STD	1.69	1.32	2.09	1.27	0.41	2.00	2.38	2.51
CV (ppm)	2.7	1.9	2.7	1.5	0.4	2.0	2.2	2.2

**Fig. 7:**
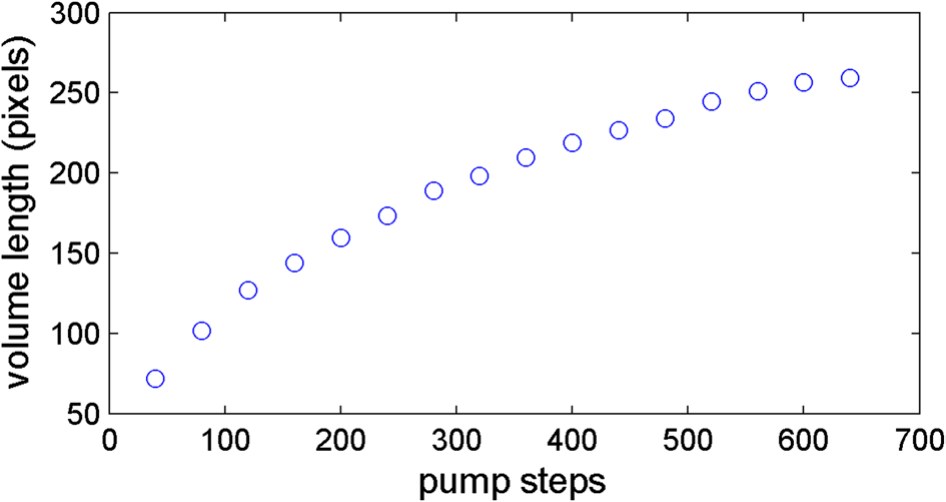
The relation between the number of pump steps and average volume length

**Fig. 8:**
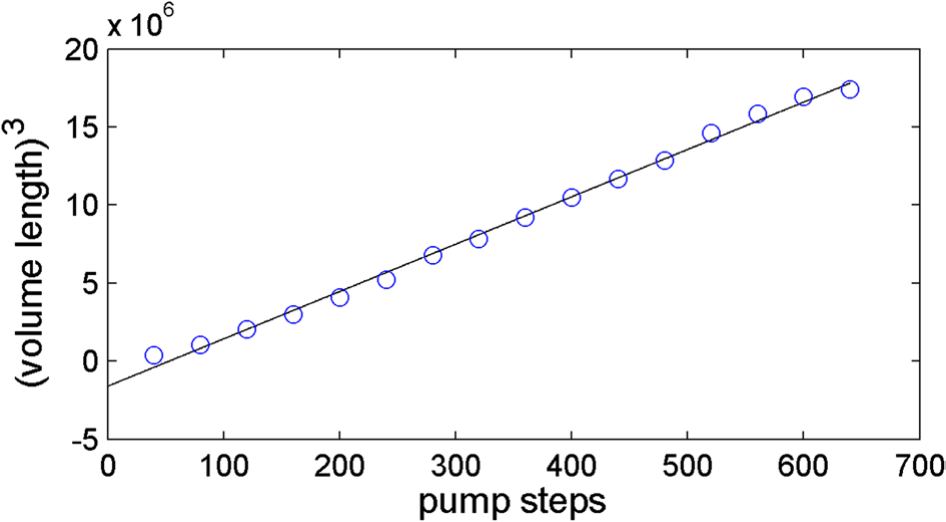
The linearity between the pump steps and cube of the average volume length (fitted linear equation: *y* = 30337*x* + 500.8, *R*^2^ = 0.996)

Table 2 shows the statistics for the relation between the pump steps and volume lengths calculated from the image processing. In accordance to Table 2, the pump showed high precision having CV values of less than 3.1% and was independent from the pump steps. Figure 7 depicts the relation between the pump steps and volume lengths. As the shape of the tip was a cone, the volume of the reagent was proportional to the cube of the volume length as shown in the figure. Therefore the relation between the pump steps and the cubes of the volume lengths were highly linear as shown in Fig. 8 (R^2^ = 0.996).

**Table 2:** The relation between the number of motor steps and volume length from image processing

Steps	40	80	120	160	200	240	280	320
Mean	72	101.6	126.8	143.8	159.6	173.6	189.2	198.4
STD	1.87	0.55	1.30	2.28	2.79	5.32	2.49	3.05
CV (ppm)	2.6	0.5	1.0	1.6	1.7	3.1	1.3	1.5

Steps	360	400	440	480	520	560	600	640
Mean	209.6	218.8	226.8	234.2	244.6	251.2	256.8	259.2
STD	2.19	2.49	2.59	2.95	0.89	1.30	1.79	2.17
CV (ppm)	1.0	1.1	1.1	1.3	0.4	0.5	0.7	0.8

The pump steps could be estimated by the linear relation shown in Fig. 8. The statistics of the estimation error is summarized in Table 3. Note that the standard deviation of the estimation of pump steps shown in the 2nd row was not from the average of the error but from the true steps shown in the 1st row. This reasoning was from the assumption that the average error might converge to zero with more experiments. As the pump step becomes larger, the standard deviation becomes larger, so it is more reasonable to investigate the pump performance by examining the relative standard deviation. The relative standard deviation is usually divided by the average of the standard deviation, but is divided into a true step instead of an average with the same reasoning as described above. The 3rd row of the table showed the relative standard deviations, and they were less than 9.4% except that for the smallest pump step. Furthermore it was less than 4.5% for the steps larger than 240 steps, which are closer to the actual sample volumes. These results demonstrate that the pump malfunction can be monitored by the proposed scheme using the camera for smart devices. Possible and simple decision of the pump malfunction can be done when the difference between the pump steps and the estimates from the volume lengths break the predefined bounds.

**Table 3:** The pump step estimation error statistics

Steps	40	80	120	160	200	240	280	320
Dev.	25.3	7.5	1.9	9.9	14.4	20.1	8.6	14.3
Rel. dev. (%)	63.2	9.4	1.5	6.2	7.2	8.4	3.1	4.5

Steps	360	400	440	480	520	560	600	640
Dev.	9.2	10.8	12.0	14.7	16.1	17.1	15.3	18.3
Rel. dev. (%)	2.6	2.7	2.7	3.1	3.1	3.1	2.6	2.9

Dev.: standard deviation from the pump steps; Rel. dev.: relative standard deviation

In our previous work, the ROI image of the tip and tip holder was binarized through a similar procedure. The binary images for the tip and the holder were separated and projected vertically and horizontally, respectively. The center position and the top of the holder were calculated from the supports of the projections as in this work. The calculated positions of the tip or the holder were very linear to the pertinent stepper motor’s positions (the coefficients of determination are 0.997 and 0.999 for the tip and the holder, respectively). The cubes of the volume lengths from the presented method in this work were also highly linear to the actual reagent volume, showing the consistency of the results with the previous work.

## Discussions

In this study, an image-based monitoring method for pumping operation was introduced for diagnostic devices with automated pipetting. The support length determined from the horizontal projection of the reagent area was a good estimate of the pump steps or reagent volume. The simple image processing which includes the reagent area separation by the binarization of a specific color image with a fixed threshold, the vertical project of the binary image, and the support length calculation of scanning the projection for non-zero pixel, was sufficient to deliver the estimate. The proposed method can be applied to the detection of malfunctions of any automatic pipette device when implemented in conjunction with previous studies of misalignment detection of the tip member and tip holder.

The proposed simple image processing method using a commercial camera sensor exhibited sufficient performance at a low cost for solving the verification problems that can occur in other POCT devices as well. Owing to the widespread use of many high-performance camera sensors, this method can be applied to other similar devices.

By applying the proposed simple image processing methods to a device with a highly controllable imaging condition, user mistakes and/or device malfunction can be prevented by detecting device failure. Especially the volume estimation method presented in this study will serve for the maintenance of the pump which is one of the most important component for the devices with automated pipetting function.

The suggested image processing using the volume verification to monitor pumping operations can be employed to devices with the inaccuracy specification of less than 5%, if the dispensing target is elongated in the vertical direction as the tip. Even if the dispensing target of the device is flat such as the microtiter, this method can still be applied if there is a long reservoir between the dispenser and the target where the volume can be measured. In case that the device has an accurate and precise dispenser (CV < 5.7 ppm) as in this work, the detection of the pumping operation malfunction will be more effective rather than trying to verify the volume. Comparison studies on the malfunction detection performance between several methods was not given in this work, since it should be measured for numerous devices in production. Note that the performance of this kind of detection method is highly related to the production yield. Therefore the applicability of the presented method should be demonstrated just prior to commercialization.

## Conclusions

In this study, an image-based failure monitoring method was introduced for POCT devices with automated pipetting. The simple image processing method using a commercial camera sensor can be detect the malfunctions of POCT devices such as the tip absence or misalignment of the tip and the tip holder. Especially the presented image processing also monitors pumping operations with the inaccuracy specification of 5%.

## Abbreviations

POCT: point-of-care testing
ALH: automated liquid handler
LOC: lab-on-a-chip
ROI: region of interest
CV: coefficients of variation
